# Supplementary material for: Cost Consequences for the NHS of Using a Two-Step Testing Method for the Detection of Clostridium difficile with a Point of Care, Polymerase Chain Reaction Test as the First Step
Source: Diagnostics (Basel). 2020 Oct 14;10(10):819. doi: 10.3390/diagnostics10100819 (PMC7602277; doi:10.3390/diagnostics10100819)
Supplement: Supplementary file 1 [file diagnostics-10-00819-s001.pdf]

diagnostics

Supplementary Material

S1. Validation for NuTH change in *C. diff* algorithm: PCR prior to Toxin Testing

Following a *C. diff* test being incorrectly tested for PCR at the same time as toxin it was noted that the toxin test was positive despite the PCR test being negative. This raised questions over the validity of results produced using the current algorithm (positive GDH followed by toxin testing and a PCR only if the toxin is proven to be negative in order to identify carriers of *C. diff*). The toxin positive mandatory surveillance list was then reviewed and it was identified that approximately 10% of all toxin positive specimens sent for ribotyping did not yield growth of the organism and could therefore be a false positive result. Reporting false positive cases could have adverse outcomes for both the patient who could be unnecessarily treated and also the Trust as national targets are set for these infections with financial and reputational implications if not met.

It was agreed that a PCR should be ran on any GDH positive specimens and only if positive, a toxin test should follow. This would ensure that any toxin positive results obtained are a result of production via the toxin gene detected by PCR. The additional cost of running a PCR test on all GDH positives specimens was outweighed by the financial cost of treating patients incorrectly and penalties for not meeting national targets.

It was agreed that the testing algorithm would be implemented prior to validation and that this would be carried out prospectively. This was to ensure that positive results obtained were proven to be accurate via molecular testing in a timely manner. *C. diff* testing was carried out using the new testing algorithm (see **Error! Reference source not found.**, below). *C. diff* toxin positive specimens may be sent for ribotyping if identified on part I or part II of a patient's death certificate or if there are a cluster of cases. The first 10 C. difficile toxin positive specimens diagnosed using the new testing algorithm, requiring ribotyping would be used to validate the testing algorithm in place. Specimens were sent to the Leeds CDRN laboratory where they were cultured for *C. diff* and subsequently ribotyped. Results were then made available on the CDRN website and the presence of an identified ribotype was used to confirm correct identification of the *C. diff* case using GDH followed by PCR (Cepheid) and then toxin testing.

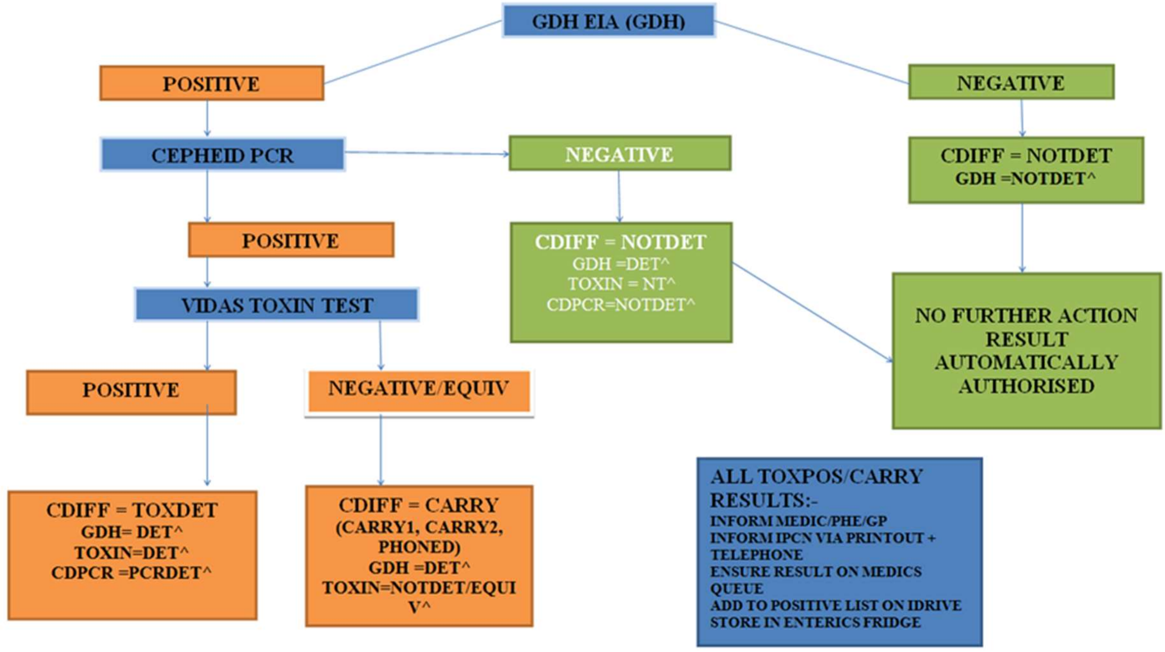

Figure S1. Novel C. diff testing algorithm under investigation in the NuTH laboratories.

10 specimens testing positive for *C. diff* using PCR on the Cepheid were confirmed to be positive by ribotyping at the Leeds CDRN:

Table S1. Comparison of Cepheid PCR results to Leeds CDRN ribotyping results.

Patient.	CT value	Result	CDRN ribotype
1	23	Toxin B positive	R020
2	23.2	Toxin B positive	R023
3	27.7	Toxin B positive	R005
4	27.9	Toxin B positive	R003
5	26.5	Toxin B positive	R870
6	25.2	Toxin B positive	R014
7	31.4	Toxin B positive	R026
8	24.5	Toxin B positive	R014
9	22.5	Toxin B positive	R057
10	23.3	Toxin B positive	R005

The new testing algorithm for C. difficile implemented on 1/8/17 is fit for purpose for the diagnosis of *C. diff*. Performing PCR prior to toxin testing ensures that any toxin positive results obtained are due to the production of toxin from genes detected. This, in turn, will reduce the possibility of false positive toxin results ensuring that patient treatment and Trust figures are accurate.

S2. Model structure

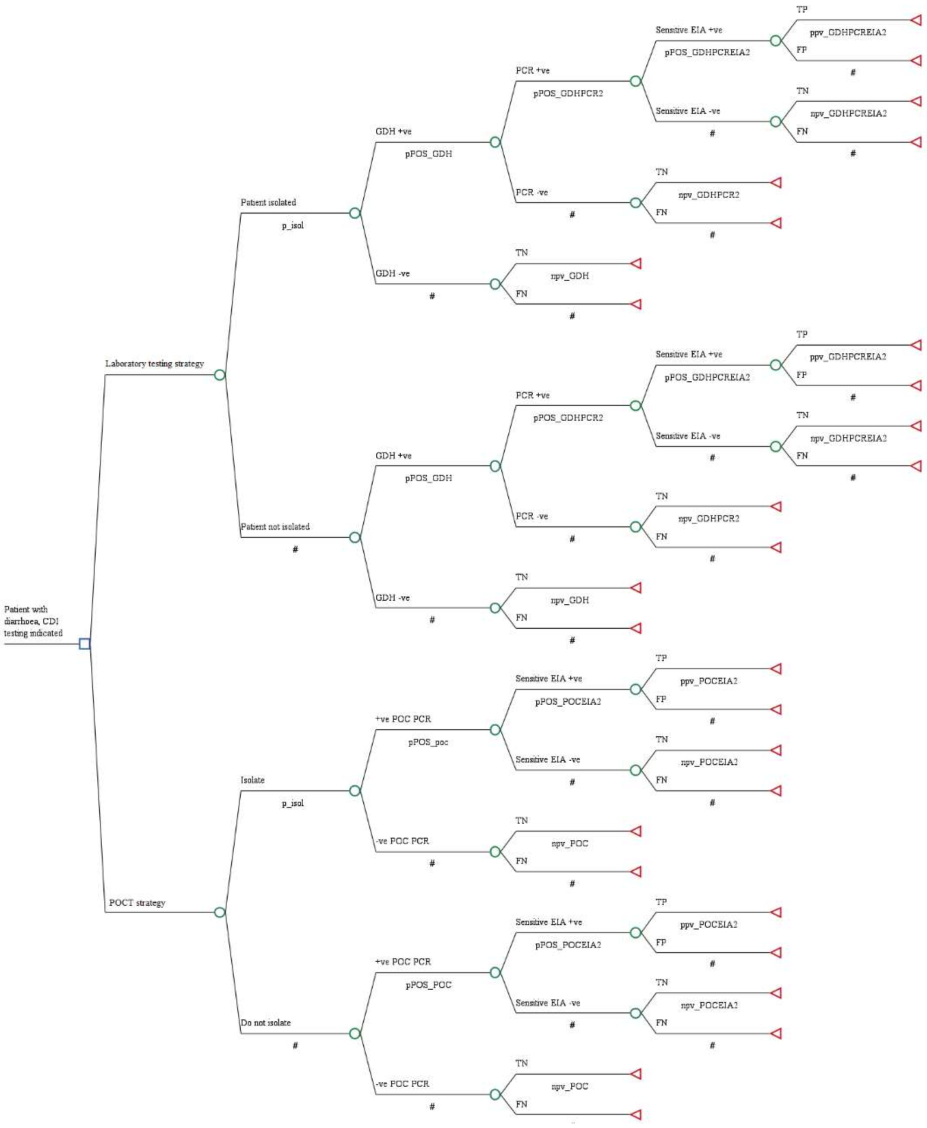

Figure S2. Simplified decision tree to assess costs and consequences of POCT strategy for CDI compared with laboratory testing strategy.

S3. Visualisations of testing algorithms

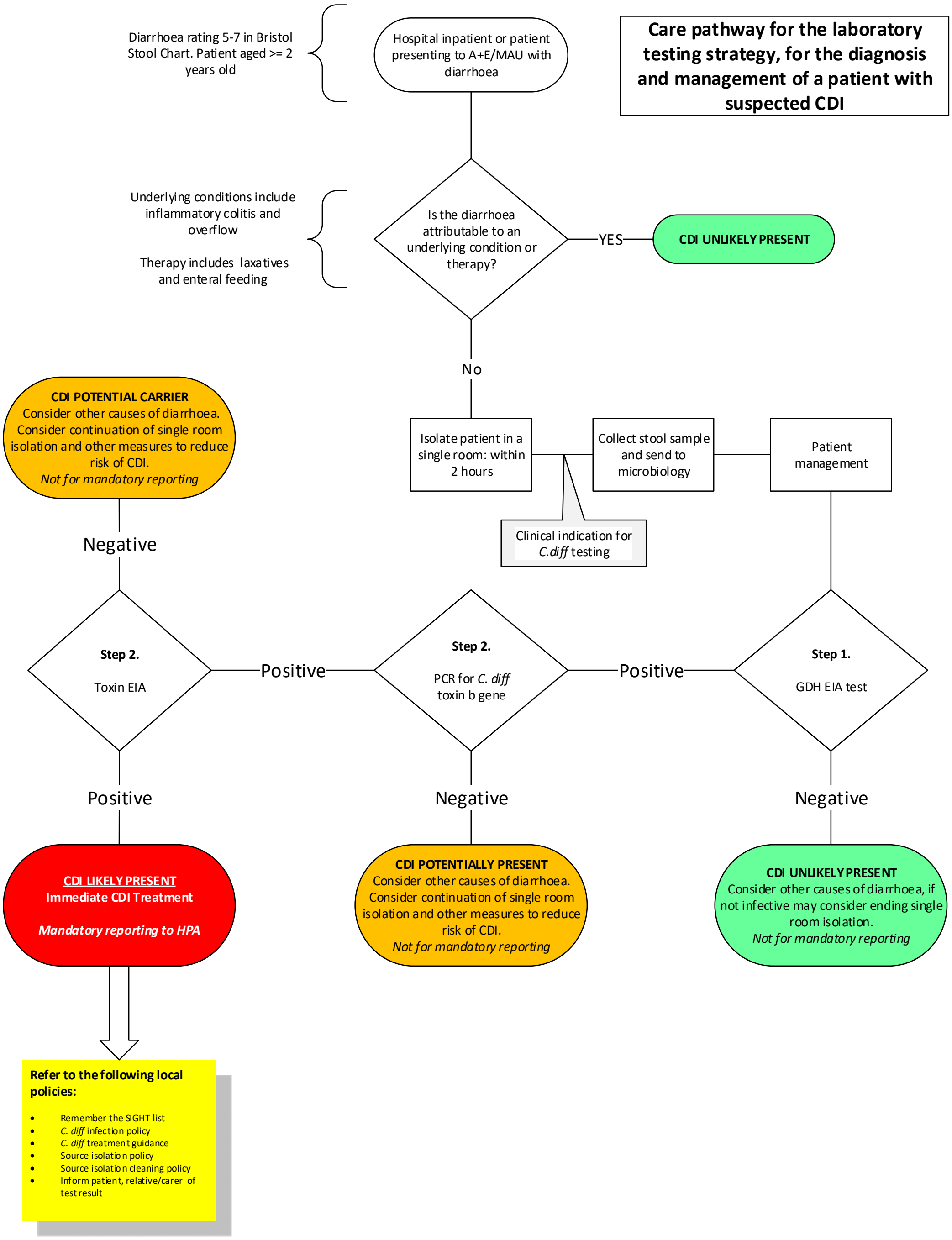

Figure S3. Care pathway of the laboratory testing strategy.

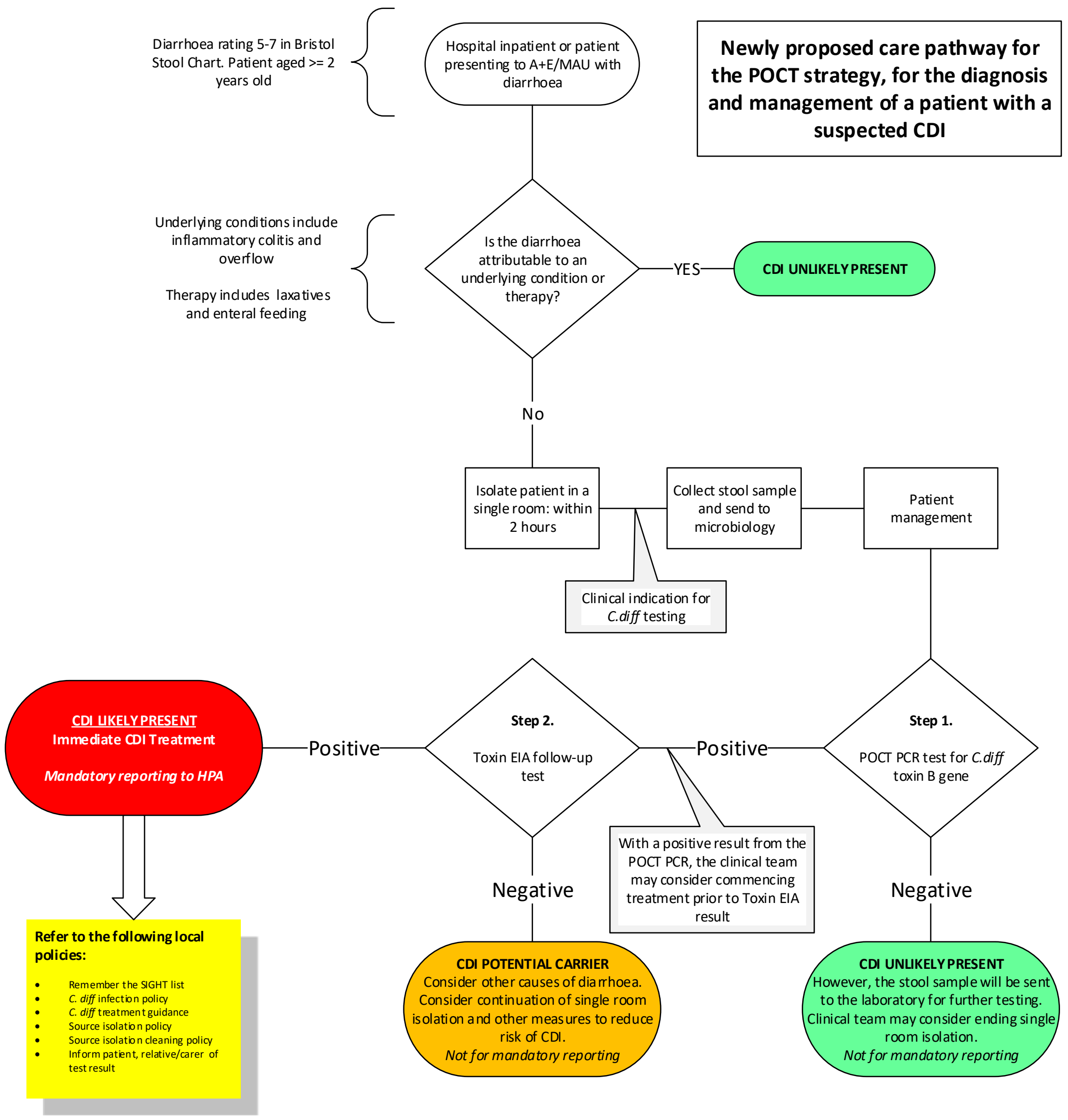

Figure S4. Proposed care pathway for the POCT strategy.

S4. Clinical interviews

Semi-structured in-depth interviews (13 in total) were conducted between January and November 2018, across the following NHS sites: Newcastle, Gateshead, Sunderland, Middlesbrough, Manchester and London. Participants were purposively selected according to their role and involvement in the diagnosis and treatment of *C. diff* and included infectious disease consultants, microbiologists, biomedical scientists, senior nurse managers and practitioners as well as Public Health England community consultants (see **Error! Reference source not found.** below, for details of distribution of roles). Two researchers (WJ and JA) conducted the fieldwork.

Table S2. Overview of interviewee roles.

Role of interviewees	n =
Infectious disease consultant	3
Consultant microbiologist	4
Laboratory-based scientist (diarrhoea/*C. diff*)	2
Public Health England (PHE) community consultants	2
Clinical Commissioning Group (CCG) director of nursing	1
Nurse matron in infection prevention and control	1

Prior to commencing this work, all necessary approvals were obtained from the Human Research Authority and the Newcastle Upon Tyne NHS Foundation Hospitals Trust R&D Committee; IRAS: 241136.

Participants were provided with information sheets in advance, and consent forms signed prior to the start of the interviews. All interviews were digitally recorded, anonymized and transcribed in full. Interviews were typically around 60 min in length and conducted on an individual, face-to-face basis or over the phone.

Transcribed interview data were analysed using thematic analysis to generate category systems and repeated themes. Emerging themes were developed in an iterative and inductive way, breaking down and reassembling the data through a coding process. Two members of the research team (WJ and JA) undertook the analysis of the interview data. These were then reviewed and discussed at wider research team meetings, with any discrepancies resolved through this process.

S5. Literature search methods

The search strategy was designed by an experienced information specialist (FB), in collaboration with the project team. It aimed to identify existing economic models addressing the diagnosis or care pathway of patients with *C. diff* or other sources of diarrhoea. We conceptualised the search as:

A: [C. diff OR diarrhoea] AND B: [economic models]

For element A we identified thesaurus headings that described C. diff and diarrhoea. We did not use title and abstract terms because 'diarrhoea' is a term that is in common usage in many irrelevant studies so it would have made the search unmanageable, and the aim was not to conduct a comprehensive systematic review. For element B we used a search filter, a search strategy that has been tested and aims to find particular type of study. We used the Canadian Agency for Drugs and Technologies in Health (CADTH) search filter for economic evaluations and selected the section focused on economic models.

We searched the following databases in March 2018 with no date limits, restricted to publications in English:

- MEDLINE (OVID) 1946 to March Week 1, 2018
- EMBASE (OVID) 1980 to 2018 Week 10

The results were downloaded to and de-duplicated in Endnote.

See **Error! Reference source not found.** below for a summary of MEDLINE search strategy results.

Table S3. MEDLINE search strategy results.

#	Searches	Results
1	Clostridium difficile/	6969
2	Clostridium Infections/	4561
3	diarrhoea/	22296
4	exp models, economic/	11654
5	economic model*.ab,kf.	1782
6	markov chains/	11422
7	markov.ti,ab,kf.	13123
8	monte carlo method/	22363
9	monte carlo.ti,ab,kf.	23612
10	exp Decision Theory/	8647
11	(decision* adj2 (tree* or analy* or model*)).ti,ab,kf.	13379
12	or/4-11	69936
13	1 and 12	26
14	2 and 12	26
15	3 and 12	89
16	13 or 14 or 15	121

17	limit 16 to humans	119
18	limit 17 to English language	112

S6. Inputs for the decision model

Table S4. Prevalence of pathogens in adults investigated for infectious diarrhoea in UK NHS hospitals.

Pathogen	Prevalence	Source
C. diff	10.3%	Freeman et al. [1]
All other pathogens	13.8%	Freeman et al. [1]

The average length of stay in hospital for patients with diarrhoea, CDI and other gastroenteritis-causing pathogens were obtained from NHS HRG data 2014/2015[2] as 4, 19 and 5 days respectively, and time to sample collection, before diagnostic testing, was estimated as 0.5 days by expert opinion (see Section S4). Transport time for the sample to reach the laboratory was extracted from 2014/2015 audit data from the NuTH, which encompasses two hospitals at separate sites (3 miles apart), served by a single microbiology laboratory situated at one of these sites. The common probabilities used in the model are shown in **Error! Reference source not found.**S5, below.

Table S5. Common probabilities used in the model. Where appropriate, ranges for sensitivity analysis are shown in brackets.

Name of parameter	Base case value	Description of parameter	Source
p_isol	0.5113<br><br>(0 – 1)	Probability of patient with diarrhoea being isolated upon presentation	Goldenberg et al. [3]
prev_cdi	0.10342<br><br>(0 – 0.25)	Prevalence of CDI	Freeman et al. [1]
prev_gi	0.13801<br><br>(0 – 0.25)	Prevalence of other diarrhoea causing pathogens	Freeman et al. [1]
t_avstay	4<br><br>(2 – 6 days)	Average length of stay in hospital for patient with diarrhoea	NHS HRG data 2014/2015 [2]
t_avstaycdi	19<br><br>(15-24 days)	Average length of stay in hospital for patient with CDI detected	NHS HRG data 2014/2015 [2]
t_avstayotherpathogens	5<br><br>(3 – 7 days)	Average length of stay in hospital for other diarrhoea causing pathogens detected	NHS HRG data 2014/2015 2]
**For patients with positive result for CDI**			
p_posCDIdis	0.047	Probability of patient testing positive for CDI being discharged immediately after testing as symptoms have resolved	Freeman et al. [1]
p_posCDIisolRx	0.953	Probability of isolating and treating patient who tests positive for CDI	Freeman et al. [1]
**For patients with other gastrointestinal pathogens**			

Name of parameter	Base case value	Description of parameter	Source
p_posdis	0.0470 (= p_posCDIdis)	Probability of patient being discharged as symptoms have resolved	Freeman et al. [1]
p_posisolRx	0.5295	Probability of isolating and treating another detected pathogen	Freeman et al. [1]
p_posisol	0.1302	Probability of isolating a patient and not treating another detected pathogen	Freeman et al. [1]
p_posisolrm	0.2933	Probability of removing patient from isolation/not isolating them and not treating other detected pathogen	Freeman et al. [1]
**For patients with true negative results for CDI**			
p_negCDIdis	0.8868	Probability the patient with no CDI detected will be discharged as symptoms have resolved	Goldenberg et al. [3]
p_negCDIisolinfect	0.0774	Probability the patient will be kept in isolation for suspicion of other infective causes of diarrhoea	Expert opinion, see Section S4.
p_negCDIisol	0.0258	Probability the patient will be kept in isolation for other, non-infective reasons	Expert opinion, see Section S4.
p_negCDIdeisol	0.01	Probability the patient will be removed from isolation	Expert opinion, see Section S4.
**For patients with false negative results for CDI**			
p_FnegCDIdis	0.0470 [=p_posdis]	Probability the patient with no CDI detected will be discharged as symptoms have resolved	Freeman et al. [1]
p_FnegCDIisolinfect	0.707	Probability the patient will be kept in isolation for suspicion of other infective causes of diarrhoea	Expert opinion, see Section S4.
p_FnegCDIisol	0.236	Probability the patient will be kept in isolation for other, non-infective reasons	Expert opinion, see Section S4.
p_FnegCDIdeisol	0.01 [= p_negCDIdeisol]	Probability the patient will be removed from isolation	Expert opinion, see Section S4.
**For isolated/non-isolated patients with false negative results for other pathogens**			
p_negdis	0.9573	Probability the patient with no pathogen detected will be discharged as symptoms have resolved	Goldenberg et al. [3]
p_negnegisol	0.0213	Probability that patient with no pathogen detected will be kept in isolation as symptoms persist	Goldenberg et al. [3]

Name of parameter	Base case value	Description of parameter	Source
p_negnegisolrm	0.0213	Probability that patient with no pathogen detected will be removed from isolation as symptoms persist	Goldenberg et al. [3]

Table S6. Diagnostic accuracy and time to result data for individual tests.

Name of parameter	Base case value	Range for sensitivity analysis	Description of parameter	Source
sens_gdh	0.96	0.86 – 0.99	Sensitivity of Glutamate dehydrogenase (GDH) enzyme immunoassay	Crobach et al. [4]
spec_gdh	0.96	0.91 – 0.98	Specificity of Glutamate dehydrogenase (GDH) enzyme immunoassay	Crobach et al. [4]
sens_pcr	0.95	0.92 – 0.97	Sensitivity of Molecular test (NAAT or PCR) for the tcdB gene	Crobach et al. [4]
spec_pcr	0.98	0.97 – 0.99	Specificity of Molecular test (NAAT or PCR) for tcdB gene	Crobach et al. [4]
sens_eia	0.57	0.51 – 0.63	Sensitivity of enzyme immunoassay test for toxin.	Crobach et al. [4]
spec_eia	0.99	0.98 – 0.99	Specificity of enzyme immunoassay test for toxin.	Crobach et al. [4]
sens_poc	0.95	0.87 – 0.99	Sensitivity of GenePOC™ CDiff assay for the tcdB gene	GenePOC™ package insert
spec_poc	0.93	0.91 – 0.95	Specificity of GenePOC™ CDiff assay for the tcdB gene	GenePOC™ package insert
t_sample	0.5 days	0 – 2	Average time to obtain sample	Expert opinion, see Section S4.
tt_tolab	0.61 days	0 - 1	Time from sample collection to laboratory	NuTH Audit data (2016-2017)
ttr_gdh	50 mins + 10 mins centrifuging = 60 mins<br><br>0.042 days	0.0315 – 0.053	Time to result for glutamate dehydrogenase (GDH) enzyme immunoassay	NuTH labs
ttr_pcr	45 mins<br><br>0.031 days	0.0235 – 0.0387	Time to result for molecular test (NAAT or PCR) for the tcdB gene	NuTH labs
ttr_eia	67 mins + 5 mins centrifuging = 72 mins<br><br>0.05 days	0.0375 – 0.075	Time to result for enzyme immunoassay test for toxin.	NuTH labs

Name of parameter	Base case value	Range for sensitivity analysis	Description of parameter	Source
ttr_poc	70 mins + 2.5 mins preparation time = 72.5 mins<br><br>0.0503 days	0 - 0.5 days	Time to result for GenePOC™ CDiff assay for presence of tcdB gene	GenePOC™ list time plus estimate

Table S7. Resource use and diagnostic testing costs. Where appropriate, ranges for sensitivity analysis are shown in brackets.

Name of parameter	Base case value (range as %)	Description of parameter	Year of estimate	Source
c_gdh	£4.50	Cost of GDH test	2017/2018	NuTH Microbiology laboratories
c_pcr	£24.75	Cost of PCR test	2017/2018	NuTH Microbiology laboratories
c_eia	£4.50	Cost of toxin EIA test	2017/2018	NuTH Microbiology laboratories
c_stapathogens	£40.13	Cost of stand GI panel	2017/2018	NuTH Microbiology laboratories
c_pocPCR	£17.82<br><br>(50 – 150%)	List price of GenePOC POC CDiff test	2018	GenePOC™ (price provided €20 –<br>€1 = £0.89, conversion rate on 15/08/2018)
c_bedday	£541.72<br><br>(50 – 150% of base case)	Cost of bed day in general adult ward	Inflated to 2017 estimate from 2015/2016 tariffs	NHS Reference Costs 2015/2016 [2]
c_isolday	£643.95<br><br>(50 – 150% of base case)	Cost of bed day in adult isolation ward	Inflated to 2017 estimate from 2015/2016 tariffs	Health Protection Scotland [5]
c_dailyclean	£11.59<br><br>(50 – 150% of base case)	Cost of daily cleaning	Inflated to 2017 from 2011 cost	Allen et al. [6]

Name of parameter	Base case value (range as %)	Description of parameter	Year of estimate	Source
c_cleanstaff	£10.43<br><br>(50 – 150% of base case)	Cost of disposables and staff time for daily clean	Inflated to 2017 from 2011 cost	Allen et al. [6]

Table S8. Treatment for causes of infective diarrhoea, with prevalence extracted from Freeman et al., 2017, where possible.

Gastrointestinal pathogen	% treated	antimicrobial	% prescribed
C. diff	100%	vancomycin, or<br>metronidazole, or<br>fidaxomicin, or	52.95%<br>42.55%<br>4.5%
campylobacter	35%	ciprofloxacin	100%
salmonella<br>shigella<br>*E coli*, shiga toxin producing<br>*E coli*, non- shiga toxin producing	45%<br>45%<br>45%<br>0%	ciprofloxacin or<br>amoxicillin	75%<br>25%
Giardia	95%	metronidazole	100%
cryptosporidium	0%	paromomycin<br>nitazoxanide	0%<br>0%

Table S9. Treatment costs.

Name of parameter	Base case value (cost per pack)	Description of parameter	Year of estimate	Source
c_vanc	£132.47	cost of oral vancomycin	2017/2018	British National Formulary [7]
c_metri	£2.48	cost of oral metronidazole	2017/2018	British National Formulary [7]
c_fidax	£1350.00	cost of oral fidaxomicin	2017/2018	British National Formulary [7]
c_ery	£1.36	cost of oral erythromycin	2017/2018	British National Formulary [7]
c_cip	£0.96	cost of oral ciprofloxacin	2017/2018	British National Formulary [7]
c_amox	£1.06	cost of oral amoxicillin	2017/2018	British National Formulary [7]

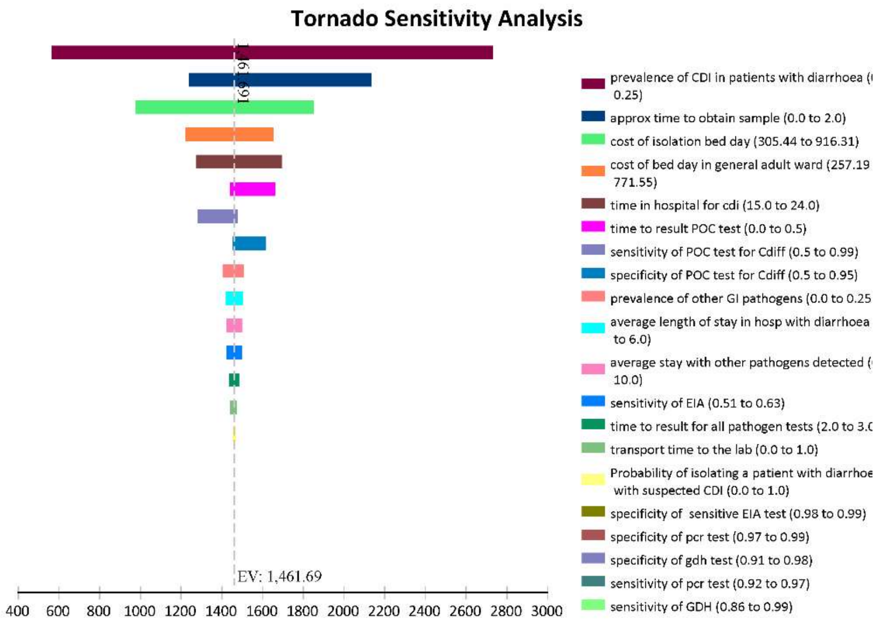

Figure S5. Tornado diagram showing the results of a univariate sensitivity analysis for variable parameters within the model. The results show the per-patient expected cost of the POCT strategy. Per patient expected cost of the POCT strategy (£)

References

1. Freeman K, Mistry H, Tsertsvadze A, et al. Multiplex tests to identify gastrointestinal bacteria, viruses and parasites in people with suspected infectious gastroenteritis: a systematic review and economic analysis. *Health technology assessment (Winchester, England)* 2017;21:1-188.
2. Department of Health. NHS reference costs 2014-15; Accessed October 22, 2018. https://www.gov.uk/government/publications/nhs-reference-costs-2014-to-2015.
3. Goldenberg SD, Bisnauthsing KN, Patel A, et al. Point-of-Care Testing for Clostridium Difficile Infection: A Real-World Feasibility Study of a Rapid Molecular Test in Two Hospital Settings. *Infectious diseases and therapy* 2014;3:295-306.
4. Crobach MJT, Planche T, Eckert C, et al. European Society of Clinical Microbiology and Infectious Diseases: update of the diagnostic guidance document for Clostridium difficile infection. *Clinical Microbiology and Infection* 2016;22:S63-S81.
5. Health Protection Scotland. *National Services Scotland, NHS Scotland MRSA Screening Pathfinder Programme - Final Report Volume 2: An Assessment of the Economics, Implementation and Modelling of Universal Screening.* 2011.
6. Allen SJ, Wareham K, Wang D, et al. A high-dose preparation of lactobacilli and bifidobacteria in the prevention of antibiotic-associated and Clostridium difficile diarrhoea in older people admitted to hospital: a multicentre, randomised, double-blind, placebo-controlled, parallel arm trial (PLACIDE). *Health technology assessment (Winchester, England)* 2013;17:1-140.
7. British National Formulary. [online; accessed 22-10-18]. 2014/2015. http://www.bnf.org.

© 2020 by the authors. Submitted for possible open access publication under the terms and conditions of the Creative Commons Attribution (CC BY) license (http://creativecommons.org/licenses/by/4.0/).
